# Absence of clinical disease and contact transmission of HPAI H5NX clade 2.3.4.4 from North America in experimentally infected pigs

**DOI:** 10.1111/irv.12463

**Published:** 2017-09-02

**Authors:** Bryan S. Kaplan, Mia K. Torchetti, Kelly M. Lager, Richard J. Webby, Amy L. Vincent

**Affiliations:** ^1^ USDA Agricultural Research Service Virus and Prion Research Unit National Animal Disease Center Ames IA USA; ^2^ USDA Animal and Plant Health Inspection Service National Veterinary Services Laboratory Ames IA USA; ^3^ Department of Infectious Diseases St. Jude Children's Research Hospital Memphis TN USA

**Keywords:** clade 2.3.4.4, H5, highly pathogenic avian influenza, influenza, North America, pigs, swine

## Abstract

**Background:**

In the fall of 2014, highly pathogenic avian influenza (HPAI) subtype H5N8 clade 2.3.4.4 was introduced into North America by migrating waterfowl from Asia where, through reassortment, novel HPAI H5N2 and H5N1 viruses emerged.

**Objectives:**

Assess the susceptibility of pigs to HPAI H5N1, H5N2, and H5N8 clade 2.3.3.3 from North America.

**Methods:**

Pigs and trachea explants were inoculated with a representative panel of H5NX clade 2.3.4.4 HPAI viruses from North America. Nasal swabs, BALF, and sera were collected to assess replication and transmission in challenged and direct contact pigs by RRT‐PCR, virus isolation, hemagglutination inhibition, and ELISA.

**Results:**

Limited virus replication was restricted to the lower respiratory tract of challenged pigs, though absent in the nasal passages and trachea cultures, as determined by RRT‐PCR in all samples. Seroconversion of inoculated pigs was detected by NP ELISA but was not reliably detected by antigen‐specific hemagglutination inhibition. Boost with adjuvanted virus was required for the production of neutralizing antibodies to assess cross‐reactivity between wild‐type avian strains. All RRT‐PCR and serology tests were negative for contact animals indicating a failure of transmission from primary inoculated pigs.

**Conclusions:**

H5NX clade 2.3.4.4 strains can replicate in the lower respiratory tract of swine upon high titer inoculation, though appear to be incapable of replication in swine nasal epithelium in vivo or ex vivo in trachea explants in culture. Infected pigs did not produce high levels of serum antibodies following infection. Collectively, our data show HPAI H5NX clade 2.3.4.4 viruses to be poorly adapted for replication and transmission in swine.

## 1 INTRODUCTION

In the fall of 2014, A/goose/Guangdong/1/1996 lineage clade 2.3.4.4 highly pathogenic avian influenza (HPAI) A viruses of the H5 subtype were introduced to the North American continent, presumably carried by infected migratory waterfowl, and subsequently isolated from wild birds in Western Canada and the Northwestern United States.^1^, ^2^, ^3^, ^4^ Following introduction to the continent, H5N8 clade 2.3.4.4 viruses reassorted with North American lineage avian influenza A viruses (IAV) producing reassortant H5N1 and H5N2 viruses. Genetically, the reassortant H5 viruses combined North American avian PB1, NP, and NA gene segments with PB2, PA, HA, MP, and NS from the Eurasian progenitor H5N8 virus.^1^, ^5^ Previous studies have shown that while North American H5NX viruses are low in virulence properties and non‐transmissible in mice and ferrets, they remain highly pathogenic for many avian species.^6^, ^7^, ^8^ As pigs have been proposed to be more permissive for replication of avian IAV compared to other mammalian species,^9^, ^10^ understanding whether H5NX clade 2.3.4.4 viruses can infect and transmit in swine is a primary concern in addressing the risk emerging IAV pose to agriculture and public health.

H1N1, H1N2, and H3N2 IAV are enzootic respiratory pathogens of domestic swine with worldwide distribution, causing mild infection of the upper and lower respiratory tract. In North America, the circulating IAV subtypes H1N1, H1N2, and H3N2 are genetically diverse products of multiple reassortments, combining the viral gene segments of avian, human, and swine influenza lineage IAV.^11^ The novel gene constellations resulting from reassortment in swine provide ample genetic diversity for the emergence of viruses capable not only of increased replication and pathogenesis in swine like the triple reassortant H3N2,^12^, ^13^ but the potential ability to infect other species, including humans, exemplified by the 2009 H1N1 pandemic.^14^, ^15^ Further, pH1N1 IAV are frequently detected as a reverse zoonotic agent, with repeated introductions into swine facilitating the replacement of classical swine lineage MP gene segment with one of pandemic lineage, in addition to vastly increasing the genomic complexity of IAV genotypes circulating in North American swine populations.^16^, ^17^, ^18^ It remains to be seen if the expanded diversity of internal gene constellations of IAV now endemic in domestic swine herds will be detrimental to agricultural production and/or human health.

To assess the ability of H5NX clade 2.3.4.4 viruses to replicate and transmit in swine, pigs were experimentally infected with representative strains from the 2014‐2015 wild bird and poultry outbreak in North America. Cohorts of naïve animals were later co‐housed with challenged pigs to assess pig‐to‐pig transmission. Additionally, to better understand the tissue tropism of clade 2.3.4.4 in swine, porcine trachea explants were infected with an expanded panel of North American H5NX viruses. Replication of IAV in the upper and lower respiratory tracts was evaluated by titration of nasal swabs and BALF and pathological analysis of lung tissue at 3 and 5 days post‐infection (dpi). Seroconversion of pigs challenged with H5NX was evaluated using the hemagglutination inhibition assay and nucleoprotein (NP) enzyme‐linked immunosorbent assay (ELISA). These data provide a comprehensive understanding of the replication, pathology, and transmission of H5NX clade 2.3.4.4 from North America in swine.

## 2 MATERIALS AND METHODS

### 2.1 Viruses

H5NX clade 2.3.4.4 viruses, A/American green‐winged teal/Washington/195750/2014 (H5N1), A/northern pintail/Washington/40964/2014 (H5N2), A/turkey/Minnesota/7172‐1/2015 (H5N2), and A/gyrfalcon/Washington/41088‐6/2014 (H5N8), highly pathogenic avian influenza virus from human A/Vietnam/1203/2004 (H5N1), human pandemic virus A/CA/04/2009 (H1N1), and North American low pathogenic avian influenza (LPAI) virus A/quail/CA/K1400794/2014 (H5N8) were propagated in the allantoic cavity of 10‐day‐old embryonated chicken eggs. The allantoic fluid of inoculated eggs was collected, and 50% egg infective dose (EID_50_) titers were determined as previously described.^19^

### 2.2 Inoculation of swine trachea explants

Swine trachea explants were derived using methods of Jones et al^20^ Trachea explants produced by punch biopsies were maintained in bronchial epithelial cell basal medium (BEBM) supplemented with SingleQuot (Lonza, Walkersville, MD, USA) on transwell inserts (Corning, Tewksbury, MA, USA). Prior to infection, explants were washed 4 ×  with phosphate‐buffered saline (PBS) then incubated for 1 hour, in triplicate per virus, with an inoculum composed of 10^5^ EID_50_ in 100 μL of infection media (BEBM w/0.5% bovine serum albumin). Following the incubation period, explants were washed three times with PBS. At 8, 24, 48, 72 hours post‐infection (hpi), 300 μL of infection media was added to the apical chamber of trachea explant containing transwell inserts and incubated for 30 minutes at 37°C followed by removal and storage at −80°C. Supernatant from each time point was titrated on MDCK cells, and 50% tissue culture infectious dose (TCID_50_) was calculated as previously described.^19^

### 2.3 Inoculation of swine

A total of 85 three‐week‐old weaned pigs were used in this study. Weaned piglets were purchased from a high‐health status herd, free of IAV, porcine reproductive and respiratory syndrome virus, and *Mycoplasma hyopneumonia*. Prior to inoculation, pigs were confirmed to be serologically negative for influenza A virus by ELISA for antibodies against IAV nucleoprotein (NP). Fifteen pigs per virus were inoculated intranasally with 10^6^ EID_50_ of virus in 2‐mL Eagle's minimal essential media (Sigma, St. Louis, MO, USA), and 5 naïve animals were housed separately to serve as non‐challenged controls. Two days post‐infection (dpi), 5 naïve pigs were introduced into the same pen, one for each virus infection group, to serve as targets for direct contact transmission. Nasal swabs from inoculated and contact pigs were collected on 1, 3, 5, and 7 dpi. Five animals from each challenge group were humanely euthanized on 3 and 5 dpi, and three pigs from each group were euthanized on 21 dpi, non‐challenged control animals at 5 dpi, and all direct contact animals at 21 dpi. Blood and whole lungs were collected during necropsy. Two animals from each group were boosted intramuscularly with live virus plus adjuvant (128 HA with Emulsigen D) at 27 dpi and again at 35 dpi with adjuvanted whole inactivated virus preparations (128 HA units with Emulsigen D). All remaining animals were euthanized and blood collected at 42 dpi.

### 2.4 Virus replication and shedding

Nasal swabs were collected at 1, 3, and 7 dpi from donor, contact, and control animals and stored in 2 mL of minimum essential medium (MEM). Whole lungs were taken from five necropsied pigs at 3 and 5 dpi and from three primary inoculated pigs and all five contact animals at 21 dpi. Lavages were performed on excised lungs using 50 mL of MEM. Broncho‐alveolar lavage fluid (BALF) was collected as previously described.^21^ Both nasal swab and BALF samples were used to assess the presence or absence of IAV via reverse transcriptase real‐time polymerase chain reaction (RRT‐PCR) as previously described.^22^ Samples positive by RRT‐PCR were subjected to virus isolation in embryonated chicken eggs.

### 2.5 Serological analysis

Seroconversion of challenged and contact animals was assessed using a commercial enzyme‐linked immunosorbent assay (ELISA) and the hemagglutination inhibition (HI) assay. Whole blood was collected, and the serum was separated by centrifugation at 290 *g* for 10 minutes. Swine serum was then diluted 1:4 with receptor destroying enzyme (RDE) (Denka‐Seiken, Tokyo, Japan) and incubated for 18 hours at 37°C. Next, RDE was heat inactivated by incubation at 56°C for 30 minutes. HI assays were performed in U bottom 96‐well plates (Corning, Tewksbury, MA) by diluting 50 μL RDE‐treated sera 1:2 in PBS, incubating with 25 μL 4 hemagglutinating units (HAU) at room temperature for 1 hour, followed by a 30‐min incubation with 0.5% turkey red blood cells at room temperature. ELISA for IAV nucleoprotein (NP) was performed using FlockChek AI MultiS‐Screen Antibody Test kit following manufacturer's instructions (IDEXX Laboratories, Inc., Westbrook, ME, USA).

### 2.6 Statistical analysis

GraphPad Prism 7 (GraphPad Software, La Jolla, CA, USA) was used for statistical analysis of RRT‐PCR data. Two‐way analysis of variance was used to compare groups. *P* ≤ .05 was considered to be statistically significant.

## 3 RESULTS

### 3.1 Viral replication in upper and lower respiratory tract and in trachea explants

**Figure 1:**
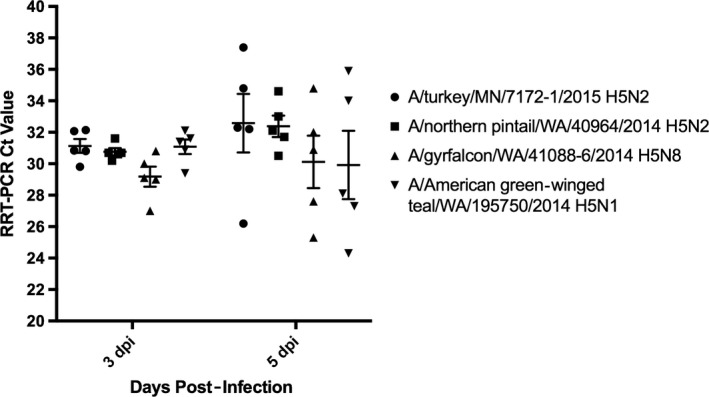
H5NX viral RNA detection in broncho‐alveolar lavage fluid (BALF) samples. RRT‐PCR Ct values were obtained from BALF collected from lungs of inoculated pigs on 3 and 5 dpi. Individual Ct values are plotted with the standard error of the mean

Nasal swabs were collected from all experimentally infected pigs on 1, 3, 5 dpi, and BALF was collected from 5 animals at 3 and 5 dpi (Table 1). Detection of IAV matrix gene was absent in all but one sample by RRT‐PCR (A/gyrfalcon/WA/41088‐6/2014 H5N8, Ct: 30.8). In contrast, all but one BALF sample were positive for IAV matrix gene by RRT‐PCR. Ct values from BALF RRT‐PCR were not significantly different between groups and time points (Figure 1). Mean Ct values from BALF collected on 3 dpi were 31.13 (A/turkey/MN/7172‐1/2015 H5N2) to 29.18 (A/American green‐winged teal/WA/195750/2014 H5N1) and 32.52 (A/turkey/MN/7172‐1/2015 H5N2) to 28.43 (A/American green‐winged teal/WA/195750/2014 H5N1) at 5 dpi. Virus isolation was attempted on BALF fluid samples, and virus was cultivated from at least one pig from all virus groups of primary inoculated pigs on 3 and 5 dpi (Table 1).

**Table 1:** Detection of H5NX influenza viral RNA or virus in the upper and lower respiratory tract and production of anti‐influenza A virus antibodies

Virus strain	NS dpi 1^a^	NS dpi 3^a^	NS dpi 5^a^	Lungs dpi 3^b^	Lungs dpi 5^b^	Seroconversion	
						HI^c^	NP ELISA^c^
A/turkey/Minnesota/7172‐1/2015 H5N2							
Primary	0	0	0	5/3	5/3	2	5
Contact	0	0	0	–	–	0	0
A/northern pintail/Washington/40964/2014 H5N2							
Primary	0	0	0	5/4	5/4	2	4
Contact	0	0	0	–	–	0	0
A/gyrfalcon/Washington/41088‐6/2014 H5N8							
Primary	0	0	0	5/5	5/3	0	3
Contact	0	0	0	–	–	0	0
A/American green‐winged teal/Washington/19750/2014 H5N1							
Primary	0	0	0	5/4	4/3	0	5
Contact	0	0	0	–	–	0	0

a Nasal swabs were tested by RRT‐PCR; Primary: n = 15 at 1 and 3 dpi and n = 10 at 5 pdi; Contact: n = 5 at each time point.

b Broncho‐alveolar lagave fluids were tested by RRT‐PCR. Positives were attempted for virus isolation in eggs; number of RRT‐PCR positive/number of virus isolation positive (n = 5).

c Number of positive serum samples at 21 dpi or 19 dpc per group (n = 5).

To further assess the ability of H5NX viruses to replicate in the respiratory tract of pigs, trachea explants were infected with an expanded panel of H5N2 and H5N8 viruses. HPAI A/Vietnam/1203/2004 (H5N1), LPAI A/quail/CA/1400794/2014 (H5N8), and A/CA/04/2009 (pH1N1) were included as control viruses. While all H5N8 and most H5N2 viruses failed to replicate ex vivo, including low pathogenic avian influenza virus A/quail/CA/K1400794/2014, the A/northern pintail/WA/40964/2014 H5N2 did replicate, though only to low titers (Table 2). Pandemic A/CA/04/2009 H1N1 and human HPAI A/Vietnam/1203/04 H5N1 were used as controls, and each virus replicated to high titers in all infected trachea explants. Together, these data show HPAI H5NX clade 2.3.4.4 H5NX viruses, unlike representative virus from an other H5N1 clades and pH1N1, to be poorly adapted for replication in the porcine upper respiratory tract.

**Table 2:** Replication of HPAI H5NX clade 2.3.4.4 viruses in swine trachea explants

Virus	Positive inserts^a^	Peak titer^b^	Time point(s)^c^
A/northern pintail/Washington/40964/2014 H5N2	–	–	–
A/gyrfalcon/Washington/41088‐6.2014 H5N8	–	–	–
A/California/04/2009 H1N1	2/2	8.5	72
A/Vietnam/1203/2004 H5N1	3/3	7	48, 72
A/quail/California/K1400794/2014 H5N8	–	–	–

–, No virus detected.

a Number of influenza positive inserts out of total number infected.

b Peak virus titer as determined by TCID_50_.

c Hours post‐infection peak titer detected.

### 3.2 Serology

Serum was collected on 21 dpi in primary pigs and 19 dpc in direct contact pigs and assayed for antibodies against IAV hemagglutinin (HA) and nucleoprotein (NP). Serum antibodies, as assessed via HI assays, were not detected in serum from pigs infected with A/gyrfalcon/WA/41088‐6/2014 H5N8 and A/American green‐winged teal/WA/195750/2014 H5N1 and in only 2 (40%, n = 5) animals from A/turkey/MN/7172‐1/2015 H5N2 and A/northern pintail/WA/40964/2014 H5N2 challenge groups. Antibodies against IAV NP were, however, detected by ELISA at an increased frequency, but not from all infected animals (Table 1).

To generate HI‐positive antisera for assay controls and to assess cross‐reactivity among the North American avian H5NX strains, 2 pigs per group were immunized via an intramuscular route with 2 doses, approximately 1.5‐2 weeks apart, of adjuvanted virus. Immune serum was collected approximately 3 weeks from the priming dose. The adjuvanted boost resulted in increased HI titers, fourfold to 16‐fold higher compared to 21 dpi titer (data not shown) with the exception of A/northern pintail/WA/40964/2014 H5N2, which did not demonstrate an increase in HI titers. Heterologous HI antibody reciprocal titers were generally within twofold to fourfold of the homologous virus titers (Table 3).

**Table 3:** Serum hemagglutination inhibition titers from H5NX clade 2.3.4.4 challenged and boosted pigs^a^

Antisera	A/turkey/MN/7172‐1/2015 H5N2	A/northern pintail/WA/40964/2014 H5N2	A/gyrfalcon/WA/41088‐6/2014 H5N8	A/American green‐winged teal/WA/195750/2014 H5N1
Antigen				
A/turkey/MN/7172‐1/2015 H5N2	**640, 160** ^**a**^	160, 320	160, 20	80, 80
A/northern pintail/WA/40964/2014 H5N2	160, 40	**40, 80**	80, 20	40, 40
A/gyrfalcon/WA/41088‐6/2014 H5N8	320, 80	80, 80	**80, 40**	80, 80
A/American green‐winged teal/WA/195750/2014 H5N1	320,40	40, 80	80, 20	**80, 160**

42 dpi sera from two pigs per exposure group immunized twice with adjuvanted whole virus due to lack of response to live virus challenge. Bold indicates homologous HI titers.

## 4 DISCUSSION

These studies were undertaken to characterize the replication and transmission kinetics of HPAI H5NX clade 2.3.4.4 viruses in swine. Experimental infection of pigs resulted in subclinical disease and lower respiratory tract restricted replication in directly inoculated animals and a complete absence of disease and evidence of replication in direct contact groups. Our data show that the HPAI H5NX clade 2.3.4.4 tested have a restricted tissue tropism in swine as evidenced by virological analysis of BALF, nasal swabs, and tissue explant samples. H5NX viruses replicated poorly in the lower respiratory tract of swine as shown by the high RRT‐PCR Ct values and inconsistent virus isolation in BALF samples. Replication was restricted to the lower respiratory tract as nasal swab samples from inoculated pigs were RRT‐PCR negative for IAV MP gene and there was a lack of viral replication on swine trachea explants. Further, inoculated pigs failed to produce even moderate titers of serum antibodies as determined by HI assay, which is atypical compared to inoculation with swine adapted IAV and some low pathogenic H5 and H7 avian IAV.^23^, ^24^

Following the emergence of HPAI H5NX clade 2.3.4.4, multiple studies have shown these viruses to be poorly adapted for replication in mammals. Kim et al^6^ found A/mallard/Korea/W452/2014 H5N8, the archetypal clade 2.3.4.4 virus, to replicate to low titers in mice, ferrets, dogs, and cats compared to other clades of HPAI H5N1 viruses. Additional studies have shown H5NX clade 2.3.4.4 viruses from North America to be capable of replication in BALB/C and C57Bl/6 mice. A/northern pintail/WA/40964/2014 H5N2 was highly lethal in BALB/C mice at an infectious dose of 10^6^ EID_50_, although this virus displayed low mortality at equal infectious doses in C57Bl/6 mice, suggesting host genetics contribute to severe disease in mammals.^7^, ^8^, ^25^ In contrast, mice inoculated with A/gyrfalcon/WA/41088‐6/2014 H5N8 at 10^6^ EID_50_ exhibited minimal weight loss (<10% initial body weight) and low mortality (80%). Similar to mice, HPAI H5NX clade 2.3.4.4 viruses exhibit low virulence in inoculated ferrets characterized by moderate infectious titers detected in nasal wash samples and are incapable of direct contact transmission to co‐housed, naïve ferrets.^7^, ^8^

Our study is the first to describe the experimental inoculation of pigs with HPAI H5NX clade 2.3.4.4 viruses. Previous studies have detailed porcine infections with viruses of A/goose/Guangdong/1/1996 clade 0, 1, 2.1, 2.2, and 2.3 H5N1 HPAI. Experimental inoculation of pigs with the first human (A/Hong Kong/156/1997) and related chicken (A/chicken/Hong Kong/258/1997) HPAI H5N1 viruses resulted in high infectious virus titers (3.6‐4.5 log EID_50_) recovered from nasal swab samples collected 2‐4 dpi, although no evidence of contact transmission was observed.^26^ More recently, Choi et al^27^ reported that pigs experimentally infected with clade 1 viruses, including the human isolate A/Vietnam/1203/2004 H5N1, displayed mild disease characterized by elevated body temperature, weight loss, and cough. Virus was detected in the nasal swab for up to 4 dpi in samples collected from inoculated animals though absent in contact animal samples. Additionally, Lipatov et al^28^ showed clade 2.1, 2.2, and 2.3 H5N1 viruses to be of low virulence in pigs, with infectious virus titers in nasal swabs and tissue samples significantly lower than from animals infected with swine H1N1 and H3N2. Although viruses used in these previous studies are representative of lineages associated with human infections and are capable of limited replication in the upper respiratory tract of swine, overall, they are avirulent in pigs consistent with our challenge data using clade 2.3.4.4 IAV.

Swine are susceptible to infection with low pathogenic avian influenza viruses (LPAI) of multiple subtypes. Multiple studies have shown pigs inoculated with LPAI exhibit little to no clinical disease and no contact transmission.^10^, ^23^ Kida et al and De Vleeschauwer et al describe pigs inoculated with LPAI shed infectious virus from the upper respiratory tract less efficiently and at lower titers than swine IAV. In contrast, a recent study examining North American H5 and H7 LPAI infection in swine found the lower lung to be the sole site of virus replication as evidenced from IAV matrix gene‐positive BALF samples.^24^ In addition to the discrepancy in IAV detection in the upper respiratory tract of avian LPAI inoculated pigs, virus detection of the North American LPAI occurred at a lower frequency and shorter duration than pigs inoculated with a control swine H1N1 and/or H3N2 virus. Virus lineage and test method could potentially contribute to the observed differences in virus detection in the upper respiratory tract. Eurasian lineage viruses may be more capable of replication in the upper respiratory tract of swine compared to avian IAV circulating in North America. Also, differences in detection by virus isolation in embryonated chicken eggs compared to RRT‐PCR between studies should be noted. Overall, results from previous challenge studies are in agreement with our data highlighting the absence of clinical disease and contact transmission as well as identifying the lower respiratory tract as the primary site of replication of avian influenza in swine. This is consistent with the relative distribution of alpha‐2,3 sialic acid receptors in the pig lung compared to pig nasal epithelium.

Ex vivo inoculation of swine trachea explants was conducted to further assess the replication kinetics of HPAI H5NX in porcine respiratory tissue. Trachea explants maintain the normal in vivo tissue architecture and sialic acid receptor distribution, abundant alpha‐2,6‐linked (human) and a complete absence of alpha‐2,3‐linked (avian) receptors.^29^, ^30^ Our findings are consistent with previous works where IAV isolated directly from avian species are incapable of replication on trachea explants. Further, human IAV of avian lineage (H5N1 and H7N9) are capable of replication in porcine tissue ex vivo suggesting avian viruses accrue molecular changes following replication in humans conferring a replicative phenotype in the upper respiratory tissue of swine.^20^, ^29^ Combined with results from in vivo challenge, these data highlight an inability of wild‐type avian isolates of HPAI H5NX to replicate in the upper respiratory tract of swine.

The data presented here demonstrate that the North American wild bird lineage H5N1, H5N2, and H5N8 clade 2.3.4.4 viruses are capable of infecting pigs by direct inoculation. Despite this, replication is restricted to the lower lung and none of the isolates were transmitted by direct contact to naïve pigs. The absence of a robust humoral immune response following challenge, as determined by HI, highlights the need for improved serological assays to ensure accurate detection of porcine infection with novel HPAI viruses should these events occur. In their current state, these data indicate that HPAI H5NX clade 2.3.4.4 viruses in North America pose little risk to the health of pigs though continued surveillance, especially in areas of domestic poultry outbreaks, is warranted.

## DISCLAIMER

Mention of trade names or commercial products in this publication is solely for the purpose of providing specific information and does not imply recommendation or endorsement by the U.S. Department of Agriculture. USDA is an equal opportunity provider and employer.
